# Developing a transit desert interactive dashboard: Supervised modeling for forecasting transit deserts

**DOI:** 10.1371/journal.pone.0306782

**Published:** 2024-07-24

**Authors:** Seung Jun Choi, Junfeng Jiao

**Affiliations:** Urban Information Lab, The School of Architecture, The University of Texas at Austin, Austin, TX, United States of America; Ningbo University, CHINA

## Abstract

Transit deserts refer to regions with a gap in transit services, with the demand for transit exceeding the supply. This study goes beyond merely identifying transit deserts to suggest actionable solutions. Using a multi-class supervised machine learning framework, we analyzed factors leading to transit deserts, distinguishing demand by gender. Our focus was on peak-time periods. After assessing the Support Vector Machine, Decision Tree, Random Forest, and K-nearest Neighbor, we settled on the Random Forest method, supported by Diverse Counterfactual Explanation and SHapley Additive Explanation in our analysis. The ranking of feature importance in the trained Random Forest model revealed that factors such as density, design, distance to transit, diversity in the built environment, and sociodemographic characteristics significantly contribute to the classification of transit deserts. Diverse Counterfactual Explanation suggested that a reduction in population density and an increase in the proportion of green open spaces would likely facilitate the transformation of transit deserts into transit oases. SHapley Additive Explanation highlighted the differential impact of various features on each identified transit desert. Our analysis results indicate that identifying transit deserts can vary depending on whether the data is aggregated or separated by demographics. We found areas that have unique transit needs based on gender. The disparity in transit services was particularly pronounced for women. Our model pinpointed the core elements that define a transit desert. Broadly, to address transit deserts, strategies should prioritize the needs of disadvantaged groups and enhance the design and accessibility of transit in the built environment. Our research extends existing analyses of transit deserts by leveraging machine learning to develop a predictive model. We developed a machine learning-powered interactive dashboard. Integrating participatory planning approaches with the development of an interactive interface could enhance ongoing community engagement. Planning practices can evolve with AI in the loop.

## I. Introduction

The creation of transit gaps with urban expansion has interested transportation planners and has been a focal point of research. Transit gaps generally occur due to the mismatch between demand and supply in the level of services [1]. Notions of “accessibility” to certain transportation uses and “capability” to use them are widely considered to quantify these issues, but analytical outcomes hugely depend on the employed materials and methods [2]. Oftentimes, the research addresses the opportunities and burdens of the “disadvantaged” communities, which generally falls into the transportation equity literature in the transportation field [3]. Historically segregated individuals and communities, including people of color and low-income households [4–6], are typically found to experience significant gaps [3].

The concept of a transit desert, which addresses the quantification of transit gaps, is similar to the concept of a food desert [1,7]. Food deserts (first termed by Cummins and Macinetyre [8]) measure relative access to nutritious foods. Jiao and Dillivan [1] applied this concept to measure the gap in mass transportation systems and coined the term ’transit desert.’ Here, a transit desert refers to a geographical location where a transit-dependent population experiences a shortage in supply to meet their demand [1].

The orientation of the transit desert is tamed by historical planning policies and practices [9]. Existing studies in transit deserts have well investigated its existence around the globe [10–12]. However, they stay at the stage of identifying transit deserts and fail to examine the underlying cause of the resulting transit deserts. It is already known that transit deserts possess a greater number of disadvantaged populations than regions where transit supply surpasses demand [12]. Still, the studies hardly address the cause of transit deserts or reform an ambiguous dialogue that there’s an issue with the supply side [13]. The results of transit desert analysis can be applied to leverage policy practices, but studies remain of a reformist orientation. A structural cause should be addressed to “transform” the practice rather than staying to “reform” the practice [2].

In the meantime, the capabilities of using transportation are different between men and women [7,14]. Women and gender minorities are prone to experiencing more challenges than men when using the transit system because they are more vulnerable to harassment [15]. The need to consider fundamental differences between the two sexes is clear, but often, transportation analysis is akin to using simplified aggregation [16]. The limitation of previous analyses on transit deserts repeats the tradition of using simplified aggregation.

The calculation of transit deserts involves classifying multiple transportation factors into demand and supply factors [1]. Both demand and supply are closely tied to the 5D attributes—Design, Density, Diversity, Destination, and Distance—in the built environment. The built environment has been known to significantly impact travel behavior and mode choices [17–21]. Leveraging the relative magnitude of demand and supply, a transit desert is defined as a location where demand is greater than supply. Conversely, a transit oasis is a term used in cases where supply surpasses demand.

Transit deserts primarily occur in downtown areas or central business districts, where major transit stations are located [1]. However, their distribution and occurrence vary across types of land use [22], which may be the result of historical land use regulation or policy practice. The practice of segregation policies, like redlining practices when interstate highways were built, has resulted in the urban disparities observed today [23]. The occurrence of a transit desert in this scenario would be the result of structurally segregating the transit-dependent population.

Studies on transit deserts have centered on major cities in the United States and European countries [1,11,22]. Lee et al. [12] extended the work by identifying transit deserts in Seoul, South Korea (S. Korea), while integrating the uniqueness of their city. By utilizing real-time floating population data that moves from place to place, they confirmed that transit deserts occur spatiotemporally in real-time. The disadvantaged population, such as low-income households receiving vouchers, was two times higher in transit deserts than in transit oases; the number of people with disabilities was also greater in transit deserts. Transit oases statistically possess more transit infrastructure than transit deserts. The identification of transit deserts aligns with investigating equity concerns in transportation and addressing the challenges of different population groups [24].

Nevertheless, studies on transit deserts remain in the identification stage [1,11–13]. The underlying causes of transit deserts remain unexplored. They reform the notion of equity in transportation rather than transform it [2].

The notion of equity first appeared during the Civil Rights Act of 1964 [25]. In the planning field, equity touches on the issue of distributive justice, as described by Rawls [26], incorporating concepts of equality and addressing the needs, demands, preferences, and willingness of people [27]. It first influenced the conceptualization of climate/environmental justice [28]. Later, reflecting on works in environmental justice concerning the disproportionate burden, transportation scholars coined the term equity in transportation. Transportation equity addresses the benefits and burdens of using transportation across different socio-demographic groups and discusses their relativeness [3].

Measuring transportation equity exists in various forms, and the results depend on the metric we use [2]. The identification of equity; “of what,” “for whom,” and “how much” is addressed in stages [3]. Generally, historically disadvantaged communities, including low-income households and communities of color, are considered [3,29]. Studies often focus on metropolitan areas, and attempts to address conditions in rural or suburban regions are being conducted [2].

The transportation equity “of whom” is likely to diverge when applying the lens of perspectives from different genders. For feminist scholars, the simple aggregation of travel behavior, without considering the uniqueness between sexes and attempts to disaggregate behavior, is pointed out to be lacking in transportation research [16]. Women’s travel behavior differs from that of men [30]. Gender minorities, including women and LGBTQ+ communities, are more prone to harassment [15]. Their safety and security impact their perceptions and actions in cities [31,32].

A threshold definition is preferably applied to address the issues of equity [2,33]. Other approaches include using a graduated scale, index, and Geographic Information System (GIS) integration [2]. The identification of transit deserts relates to previous approaches, respectively. The transit desert evaluates the accessibility of transit, as the number of certain transit stations is included in the supply factors. Measuring accessibility to specific transportation infrastructure or programs is a key concern in transportation equity [34], which discusses different human capabilities in using transportation [29]. Transit deserts use the aggregation of multiple variables in regions. The setting would be more nuanced if the measurement of capabilities were more oriented toward regional capabilities.

Overall, the transportation equity framework is quite solid and effective at addressing issues in transportation. However, some scholars have critiqued that there’s a habit of perpetuating tradition in the form of paying lip service to the notion of equity [3,35]. As an alternative, Karner et al. [3] introduced a "justice" framing, suggesting a disentanglement of planning practices with a society-centric oriented practice. Community leaders and local grassroots organizations are suggested as stakeholders to truly transform transportation planning practices. The manner and extent to which planning practitioners communicate with local stakeholders are critical to actualizing the notion of justice.

Our analysis of transit deserts is rooted in transportation equity. Given that transit deserts address the burdens of disadvantaged groups, they consider those groups’ transportation needs by quantifying regional capabilities. We expand the work by disaggregating the demand factors according to different sexes to broaden the equity “of whom” in transit deserts. Moreover, we investigate the underlying causes of transit deserts to suggest *actual* means to mitigate them. We present a supervised modeling framework for forecasting transit deserts to examine factors associated with the cause of transit deserts with the validation of our presented model. Recently identified characteristics of transit deserts are applied. Our studies propose ways to utilize the investigation of transit deserts to inform communities, ensuring that the analysis of transit deserts and transportation equity is genuinely used for practical purposes in the field, moving from equity to justice in the future. We leverage implications usable so that practitioners get a glimpse of not only identifying transit deserts but *actually* making use of them.

In summary, the limitations of existing studies are as follows: Transit desert analyses have largely stayed at the identification stage, repeating a reformist orientation. The causes of transit deserts remain unexplored. There is uncertainty about how identification from transit desert analyses can be effectively applied in planning practices. Transportation equity literature, including transit desert analysis, often relies on aggregated data, failing to clarify the differences in travel behavior between men and women. The advancement of machine learning techniques, combined with easy access to emerging empirical data sources, can bridge the existing knowledge gap. We synthesize these elements through the following research contributions:

- We integrated transit desert analysis with multi-class supervised machine learning to demonstrate potential planning interventions through forecasting and the creation of an interactive dashboard.
- In the identification of transit deserts, we disaggregated the demand factor by gender, comparing men and women, to illustrate how results differ from conventional aggregation.
- We investigated the causes of transit deserts using feature estimation modules, suggesting alternatives for modifying transportation supply factors, including attributes of the built environment.

The remainder of this article is organized as follows. First, our study presents employed materials and methods. Then, the analysis results are presented. They are further discussed with a demonstration of the interactive dashboard for planning practice. Lastly, we conclude by leaving out the limitations of our study and directions for future research.

## II. Materials and methods

### 2.1. Study area

The study area is Seoul, the capital of S. Korea, encompassing about 605 km^2^. As of 2023, the registered census population in Seoul is 9,668,008. Although the area of Seoul is smaller than that of New York City, its population density is 2.4 times greater, making it a compact metropolitan region [12]. Considering the population influx from suburban regions for work, school, and recreational activities, the population density becomes even greater. The city comprises 25 districts (locally termed “gu”) and 424 administrative boundaries (locally termed “dong”). Seoul hosts three central business districts: the Central Business District (CBD) near the city hall, the Yeouido Business District (YBD), analogous to Wall Street in the U.S., and the Gangnam Business District (GBD), known for its mega retail stores. The Han River horizontally bisects the city.

**Fig 1:**
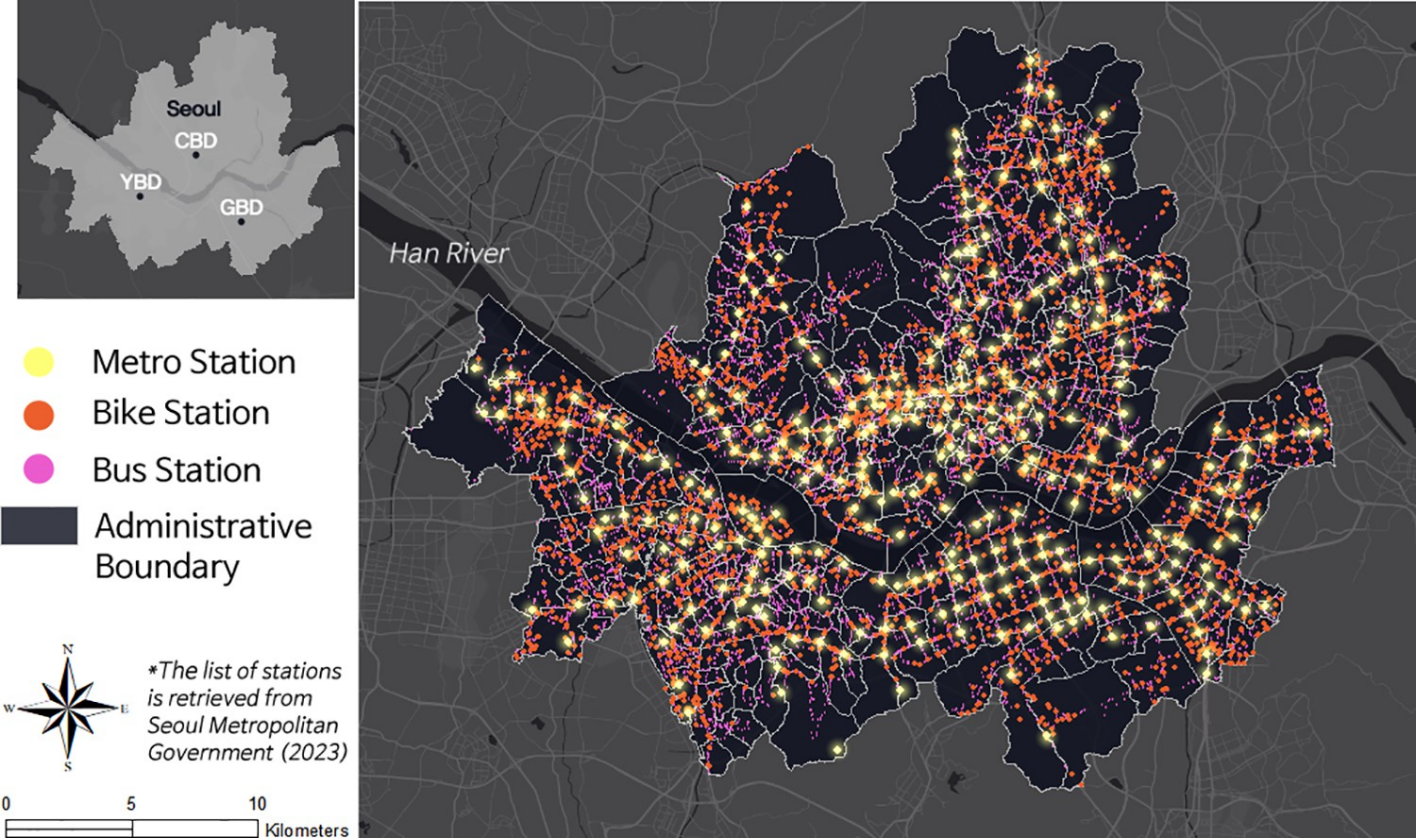
Study area and distribution of public transit stations.

The Seoul Metropolitan Government actively advertises its transportation network, which utilizes the subway, city bus, and public bike-sharing system. Public transit users can receive a discount when transferring between different transit modes. The deployment status of public transit stations in 2023 and an illustration of the study area are shown in Fig 1.

### 2.2. Identification of transit deserts

Our study conducted supervised multi-class machine learning classification modeling to forecast transit deserts. While traditional programming requires input to generate the corresponding output, supervised machine learning necessitates a set of both input and output simultaneously to create the program [36]. Therefore, the initial phase of our study first identifies transit deserts. Based on the previous calculation of transit deserts [1], we collected demand and supply factors and standardized each variable into two factors for the Z-score. This score is calculated by subtracting the population mean from the observation and dividing it by the population standard deviation. Subsequently, we took the averages of the two factors, respectively, to calculate the final Z-score. Then, by subtracting the final Z-score of the supply factor from the final Z-score of the demand factor, we can calculate the transit gap. A positive score indicates that transit demand is less than supply, while a negative score refers to cases where demand is greater than supply. A final score less than -1 is defined as a transit desert, and a final score greater than 1 is defined as a transit oasis. The following calculations were used.

$$T_{g}=T_{s}-T_{d}$$ (1)

$$Classification\;of\;Transit\;Gap=\left\{ \begin{array}{c} Transit\;Desert:T_{g}\leq -1\\ N/A:-1<T_{g}<1\\ Transit\;Oasis:T_{g}\geq 1 \end{array}\right.$$ (2)

Where *T*_*g*_: Transit Gap; *T*_*s*_: Final Z-score of Transit Supply Factor; *T*_*d*_: Final Z-score of Transit Demand Factor

The identification of transit deserts occurs in three stages. Initially, under the assumption that transportation opportunities are equally shared, the first identification employs aggregated demand across genders. The second and third identifications disaggregate the demand: the former focuses on the demand of men, while the latter centers on the demand of women. Variables for demand and supply factors are assembled based on the methods and materials explained by Lee et al. [12]. We set the study period to 2022 and preprocessed the associated big data within administrative boundaries. Our study formulated a new data frame, combining 424 administrative boundaries with 24 hours to ensure the inclusion of all 10,176 samples. Pandas [37] was utilized in the Python environment, and ArcMap 10.8 was employed to preprocess spatial information.

Demand factors considered transit-dependent populations and the floating population, with the final Z-score giving equal weight to the two. The transit-dependent population is defined by subtracting the registered vehicle numbers in 2022 from the census population aged between 20 and 84. The floating population is calculated based on human dynamics data provided by the Seoul Metropolitan Government, which has been collaborating with a mobile phone company. The data represents the population count based on mobile phone signals as people move from one place to another. It accumulates during afternoon hours, with higher volumes during weekdays compared to weekends, and decreases during holidays due to leave, confirming its temporal variations [12]. The dataset is provided with a date and hour, along with a unique ID for administrative boundaries, and is disaggregated by gender. Therefore, the initial aggregated demand uses all the records. The calculation of demand for men and women separately uses data corresponding to the sexes in both human dynamics data and the census population. We preprocessed the 2022 records to a monthly hourly average for each administrative boundary.

Supply factors primarily address the 5D attributes in the built environment, which include real-time transit usage. Destination accessibility is represented by public transit use [21]. Our study took into consideration the monthly hourly average use of metros, buses, and bike-sharing. The data is publicly available from the Seoul Metropolitan Government, and each transit station possesses a unique ID. We spatial joined the geographic information of the administrative boundary to the station in GIS and merged this information back into the raw records. After calculating the mean for each station for each month and hour within the corresponding administrative boundary, we grouped some data per month and hour based on the unique ID of the administrative boundary. These steps ensure that we obtain the monthly hourly average at the regional level. Records for metro and bus usage include the number of boarding and alighting passengers, while bike-sharing usage is recorded as trip counts for each station.

For the diversity attribute, we calculated land use entropy, referencing multiple studies [19–20,38]. Land use entropy was computed based on the ratio of land use codes per region, utilizing a land use map retrieved from the Ministry of Environment Korea. We determined the ratio of various land use types: residential, industrial, commercial, recreational, transportation, public, agricultural, and green open space to the total area of the region, employing GIS for each administrative boundary. The following equation exemplifies the calculation of land use entropy.

$$ENT=\frac{-[\sum_{j=1}^{k}p^{j}\times \ln\left(p^{j}\right)]}{\ln\left(k\right)}$$

Where ENT is land use entropy index; *p*^*j*^ refers the ratio of land use j in the region; and k is the total number of land use types in the region, k ≥ 2.

Additional design attributes considered include road ratio, number of bike routes, bike route distance, number of parking lots, and number of parking spaces [17,18]. The density attribute took into account population density [18], which refers to the number of the census population divided by the area. Distance to transit considered the number of respective transit stations, their total, and the number of city buses and taxis [19,20].

Finally, it is known that transit deserts exist spatiotemporally in Seoul [12]. We focused on peak-time periods, comprising hours from 7 to 10, 12 to 15, and 18 to 24, totaling 12 hours. We filtered the final data frame based on hours in the peak-time period to identify transit deserts. Descriptive statistics are provided as an appendix S1 Table.

### 2.3. Multi-class machine learning classification modeling

After identifying the transit deserts in three distinct scenarios: (1) using aggregated demand, (2) accounting for men’s specific demand, and (3) considering women’s disaggregated demand, the output classes (0, 1, and 2) from the aggregated analysis were reintegrated into the model to facilitate a multi-class machine learning classification forecast. The three values are determined based on the equation defined earlier, which illustrates the classification of the transit gap. A transit gap calculation below -1 is defined as a transit desert, assigned the classification label 0. A calculation above 1 indicates a transit oasis, receiving the classification label 2. Cases that do not fall into either category are labeled 1. This labeling sequence facilitates an evaluation of how the employed features positively or negatively relate to the outcome of the classification. Instances from the disaggregated demands were subsequently employed to identify unique samples that seemed to be specifically associated with a particular gender. The definitions for the utilized multi-class are as follows:

$$Multi-class\;Classification=\left\{ \begin{array}{c} 0:Transit\;Desert\\ 1:N/A\\ 2:Transit\;Oasis \end{array}\right.$$

The same variables from the initial investigation were reused as input along with the identified output. Land use variables, previously employed to calculate land use entropy, were incorporated to address the design aspect of the built environment. Regional socio-demographic characteristics such as age and the number of marginalized groups, including the elderly living alone, low-income households, and residents with disabilities, were also included. This approach is based on the understanding that transit deserts often correlate with marginalized demographics. Socio-demographic characteristics can determine whether an area is classified as a transit desert or an oasis [12].

We utilized multi-class machine learning classification models, which commonly encompass Support Vector Machine (SVM), Decision Tree (DT), Random Forest (RF), and K-nearest Neighbor (KNN). SVM, DT, RF, and KNN are typical ensemble learning methods. Ensemble learning involves using multiple models, instead of relying on a single learner for predictions. The general idea is that a collective decision from multiple models performs better than an individual decision, mirroring the human decision-making process. SVM works by finding the hyperplane that best separates different classes in the feature space. The goal is to maximize the margin between the closest points in the classes. DT possesses a tree-like structure. Nodes of DT represent tests on an attribute, branches represent the outcome of those tests, and leaf nodes represent the final decision or class. RF applies the concept of bagging (also known as bootstrapping) by building each decision tree on a random subset of the data and making decisions by averaging the outcomes of all the trees. KNN is an instance-based learning algorithm where the class of a sample is determined by the majority class among its k-nearest neighbors. Among the four methods, RF is distinguished by its use of the bagging approach. In bagging, classifiers, although all employing the same algorithm, are trained on diverse subsets of data, each obtained through unique sampling techniques.

Nevertheless, it’s challenging to definitively favor one model over another since their outcomes are contingent upon hyperparameter configurations and optimization. Hyperparameters are the configuration settings instrumental in building machine learning models. While many planning scholars have made use of these models, they rank them differently [39–47].

In our study, we employed four multi-class models. We optimized them using a technique that randomly sampled the training and validation datasets ten times [42]. The dataset was divided as follows: 60% for training, 20% for validation, and 20% for testing. We utilized a stratified strategy to ensure equal distribution of classes across training, validation, and test sets. A random seed number of one was chosen in the initial phase to maintain consistency in validation test sets across all four models. The optimization process solely relied on the training and validation data.

We sought the best settings that surpassed mean accuracy throughout the repeated validation of multiple hyperparameter combinations. For SVM, we validated varying penalty parameters of the error term (c) values (0.1, 1, 10, 100) and gamma values (1, 0.5, 0.1, 0.001) with an RBF kernel function. For DT, we considered gini and entropy criteria, best and random splitter types, tree max depths ranging from N/A to 50, min samples split from 100 to 500, and min samples leaves ranging from 1 to 50. The RF validations included tree max depths ranging from N/A to 50, min samples split from 100 to 500, and min samples leaves from 1 to 50, all with 100 estimators using a Gini criterion. For KNN, we validated neighbors of 3, 5, 7, 9, and 11, weight configurations of uniform and distance, and metrics including Euclidean, Manhattan, and Minkowski. Based on our identified hyperparameters, we tested both the scaling method and the Synthetic Minority Oversampling Technique (SMOTE) to determine if either would enhance model accuracy. The results indicated an improvement in accuracy only for SVM when using SMOTE. All models were sourced from the scikit-learn library [48]. The optimal configurations for the various models are as follows:

- For SVM, the best settings were: c = 10, gamma = 0.001, and kernel = rbf.
- For DT, the optimal parameters were: criterion = gini, max depth = N/A, min samples leaf = 10, min samples split = 100, and splitter = best.
- RF performed best with: criterion = gini, max depth = 40, min samples leaf = 1, min samples split = 100, and number of estimators = 100.
- For KNN, the optimal configuration was: metric = Manhattan, number of neighbors = 7, and weight = distance.

Using multi-classes, we evaluated the final performances of the models using macro scores. This evaluation encompasses accuracy, precision, recall, and the harmonic mean of precision and recall, known as the F1 score. The respective formulas for each score are as follows:

$$Accuracy=\frac{TP+TN}{TP+TN+FP+FN}$$

$$Precision=\frac{TP}{TP+FP}$$

$$Recall=\frac{TP}{TP+FN}$$

$$F1\;Score=2\times \frac{Recall\times Precision}{Recall+Precision}$$

Where TP: True Positive; TN: True Negative; FP: False Positive; FN: False Negative

We consistently used the same dataset for all models in the testing phase. The models were ranked in performance as RF, DT, KNN, and then SVM, as detailed in Table 1. RF achieved the performance with an accuracy of 0.974, a precision of 0.991, a recall of 0.846, an F1 score of 0.908, and 26 misplaced samples. RF surpassed DT in all metrics except for recall. Given that RF reported the fewest misclassified samples and that its F1 score was higher than that of DT, our study chose the RF model to investigate the impact of various factors on the likelihood of an area being classified as a transit desert or transit oasis.

**Table 1:** Computation of different multi-class models using testing dataset.

	SVM	KNN	DT	RF
Accuracy	0.890	0.965	0.969	0.974
Precision (Macro)	0.630	0.946	0.919	0.991
Recall (Macro)	0.340	0.819	0.870	0.846
F1 (Macro)	0.326	0.873	0.894	0.908
Misplaced Samples	112	36	32	26

**Fig 2:**
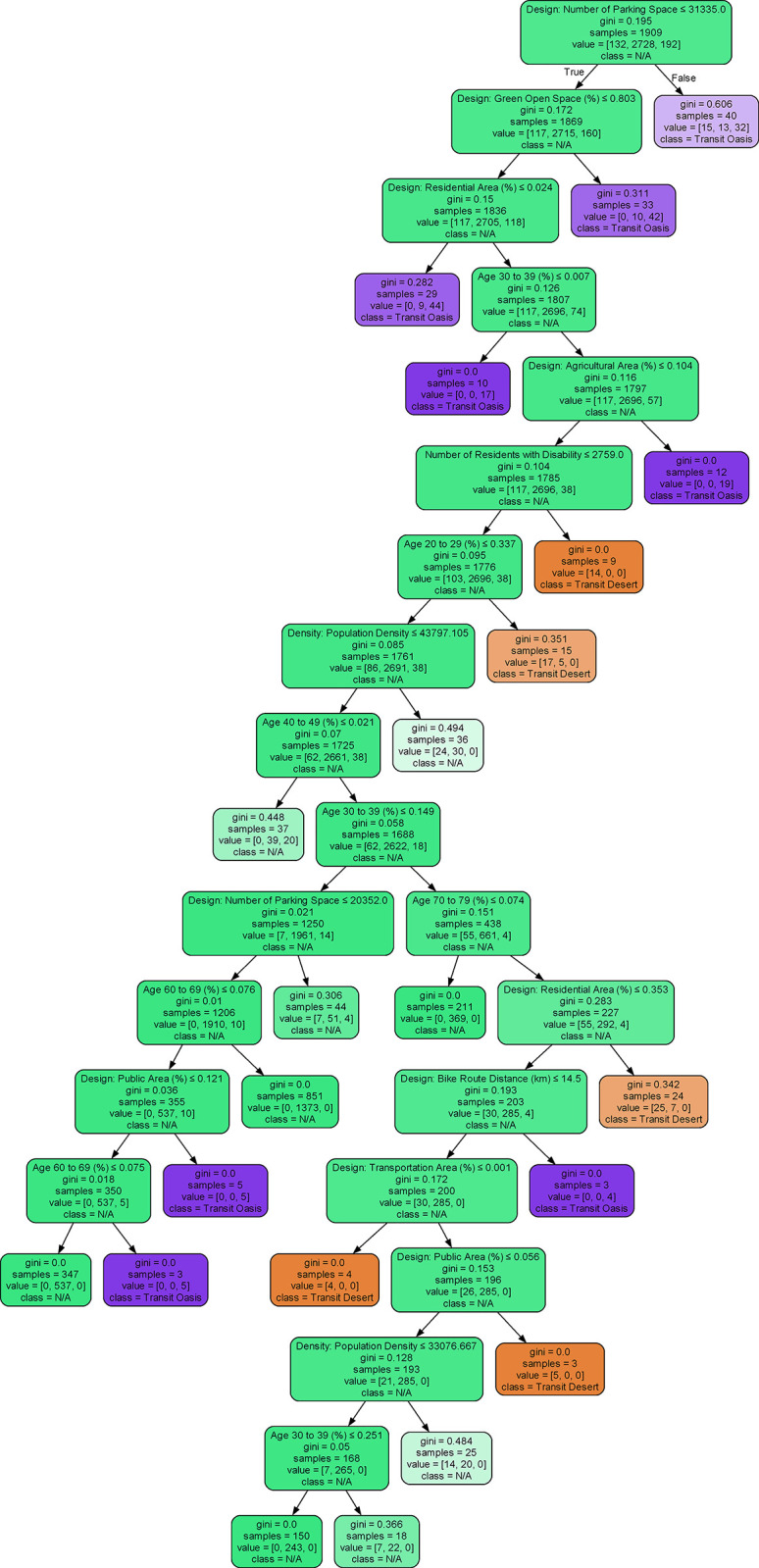
Sample of estimator in selected and trained Random Forest mode.

Fig 2 showcases a sample estimator from the chosen RF model. The samples, categorized as transit desert, N/A, and transit oasis, are depicted in distinct colors, highlighting the interplay of multiple variables in the classification process. Generally, the Gini impurity decreases as we traverse the tree from the root to the leaf nodes. A higher Gini impurity indicates a greater mix of sample types. As illustrated in Fig 2., the root node at the top splits based on the number of parking spaces being less than or equal to 31,335. Subsequent splits involve features like green open space and residential areas, showing their influence in the classification process. By following the path of splits, we can trace how a particular sample is classified.

### 2.4. Methods

**Fig 3:**
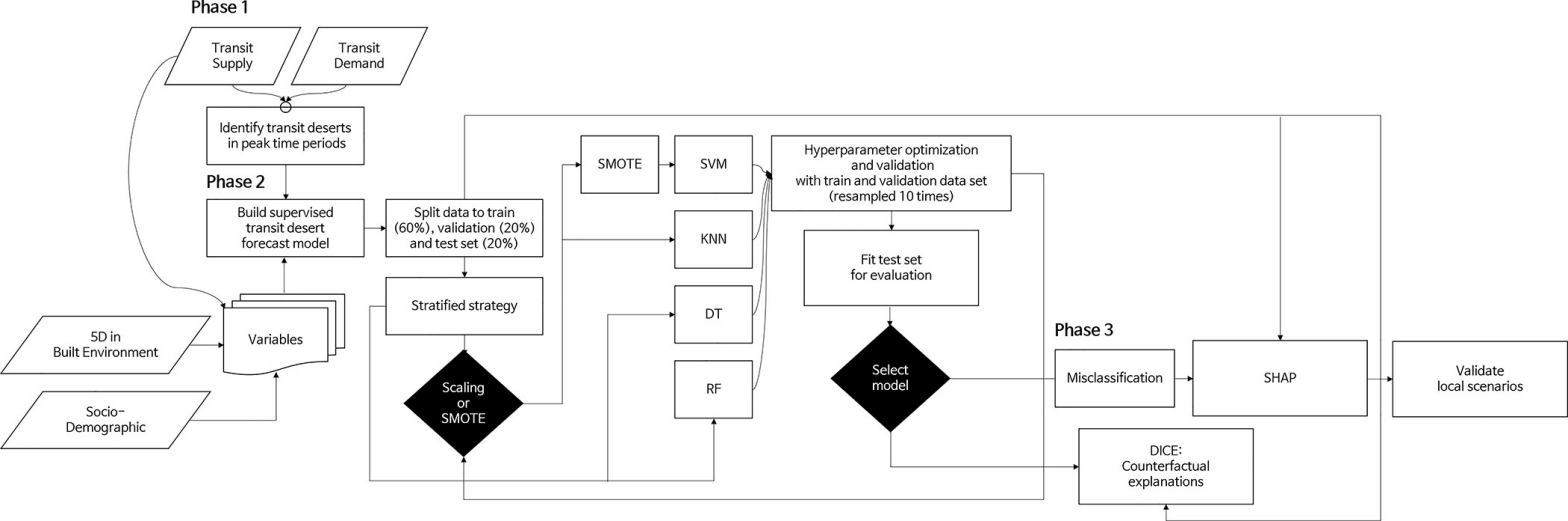
Flow chart of supervised modeling for forecasting transit deserts and validation.

A flow chart detailing the combination of methods used throughout the study is illustrated in Fig 3. In Phase 1, we investigated transit deserts during peak-time periods to identify output classes. In Phase 2, we used the input, output, and factors associated with transit deserts to construct a multi-class forecasting classification model. This model underwent a validation and optimization process, comparing it with multiple other models to select the best-performing one.

In the final phase, Phase 3, we examined the feature importance of the selected model. We investigated its feature estimation using the Diverse Counterfactual Explanation (DICE) and SHapley Additive exPlanations (SHAP) methods. DICE, a tool Microsoft offers on GitHub [49], provides "what-if" explanations for a model’s output by illustrating how changing values of independent variables affect outcomes. To cite an example from their work [49], if we were assessing eligibility for a loan, DICE might indicate that the loan would have been approved if certain circumstances had changed to a specified degree. We use an analogy to contrast cases of transit deserts with potential transit oases.

The SHAP method, introduced by Lundberg and Lee [50], addresses the interpretation of machine learning models using cooperative game theory. This method allows us to understand specific features’ positive or negative impacts on the output, offering a unique solution. To simplify the interpretation of the selected model, misclassified samples, either as transit deserts or transit oases, are presented in a Decision Plot. It provides insights into how different features influence the model’s predictions, starting from a base value. Furthermore, instances of transit deserts occurring uniquely for men or women were integrated into SHAP’s waterfall plot.

## III. Results

### 3.1. Transit desert in peak-time period

**Fig 4:**
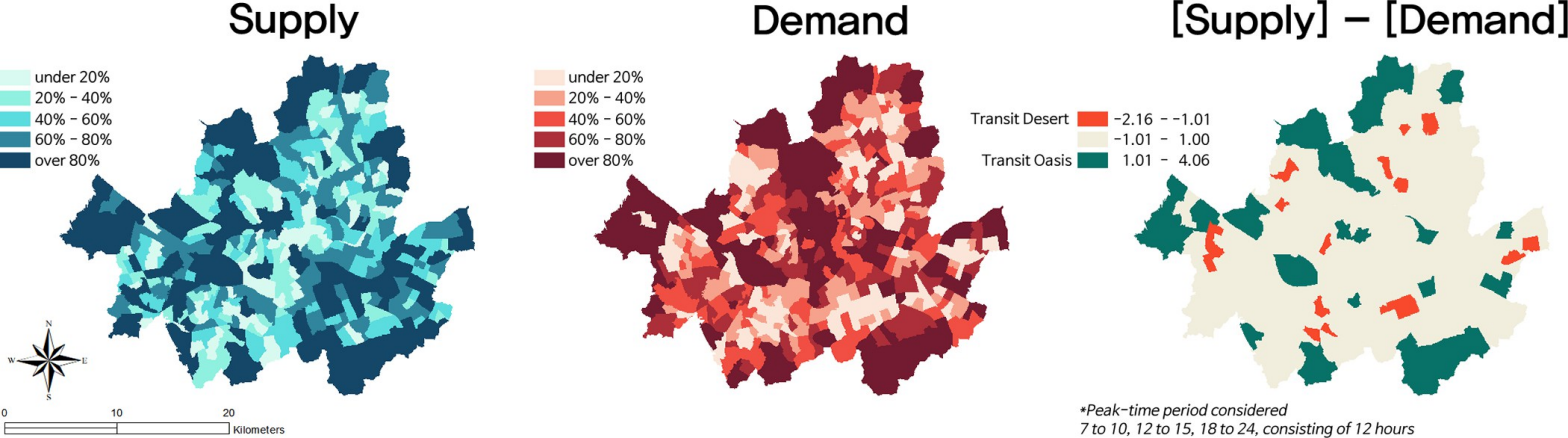
Identified transit gap in peak-time period using aggregated demand.

**Fig 5:**
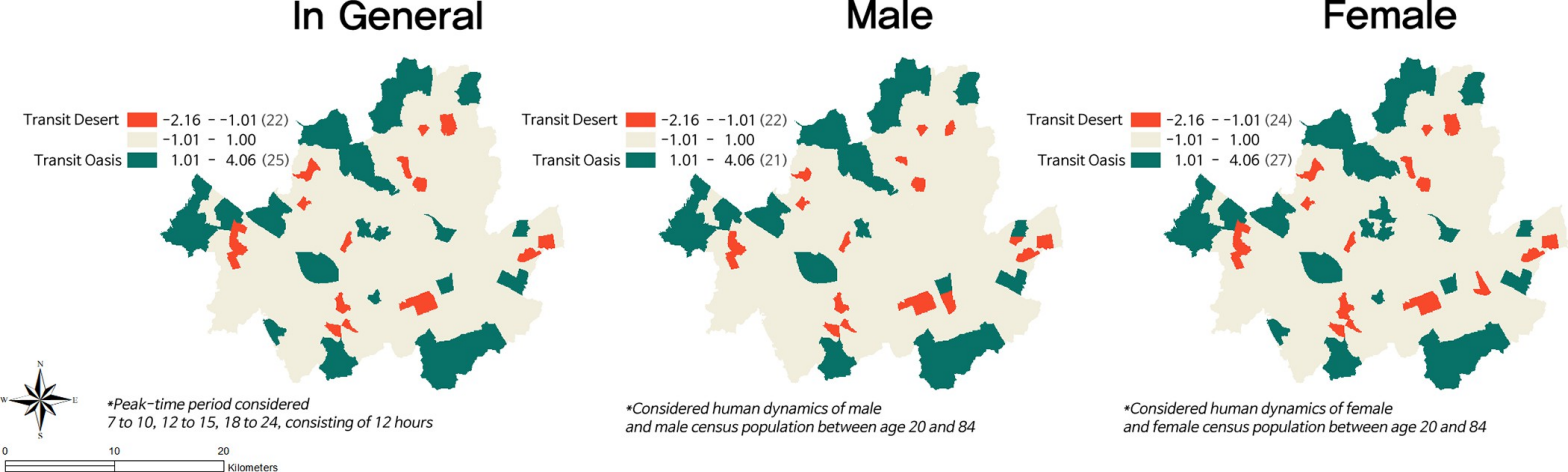
Transit gap in peak-time period using aggregation and disaggregation by sex.

Fig 4 displays the transit deserts determined by aggregated demand during peak-time periods, with corresponding final supply and demand Z-scores organized into five quantile breaks. The analysis identified 22 transit deserts and 25 transit oases in Seoul during peak times. Fig 5 showcases transit deserts discerned using disaggregated demand between genders during the same time periods, along with the corresponding counts of areas classified as either transit deserts or transit oases. Generally, aggregated and disaggregated demands pinpointed similar spatial distributions of transit deserts and oases. However, there are distinct areas that are uniquely associated with specific genders. When relying on disaggregated demand for males, the mismatch in the total number of transit deserts and oases was the smallest, reporting 22 transit deserts and 21 transit oases. Conversely, the largest mismatch occurred when using disaggregated demand for females, resulting in 24 transit deserts and 27 transit oases.

### 3.2. Selected and trained multi-class Random Forest classification model

**Fig 6:**
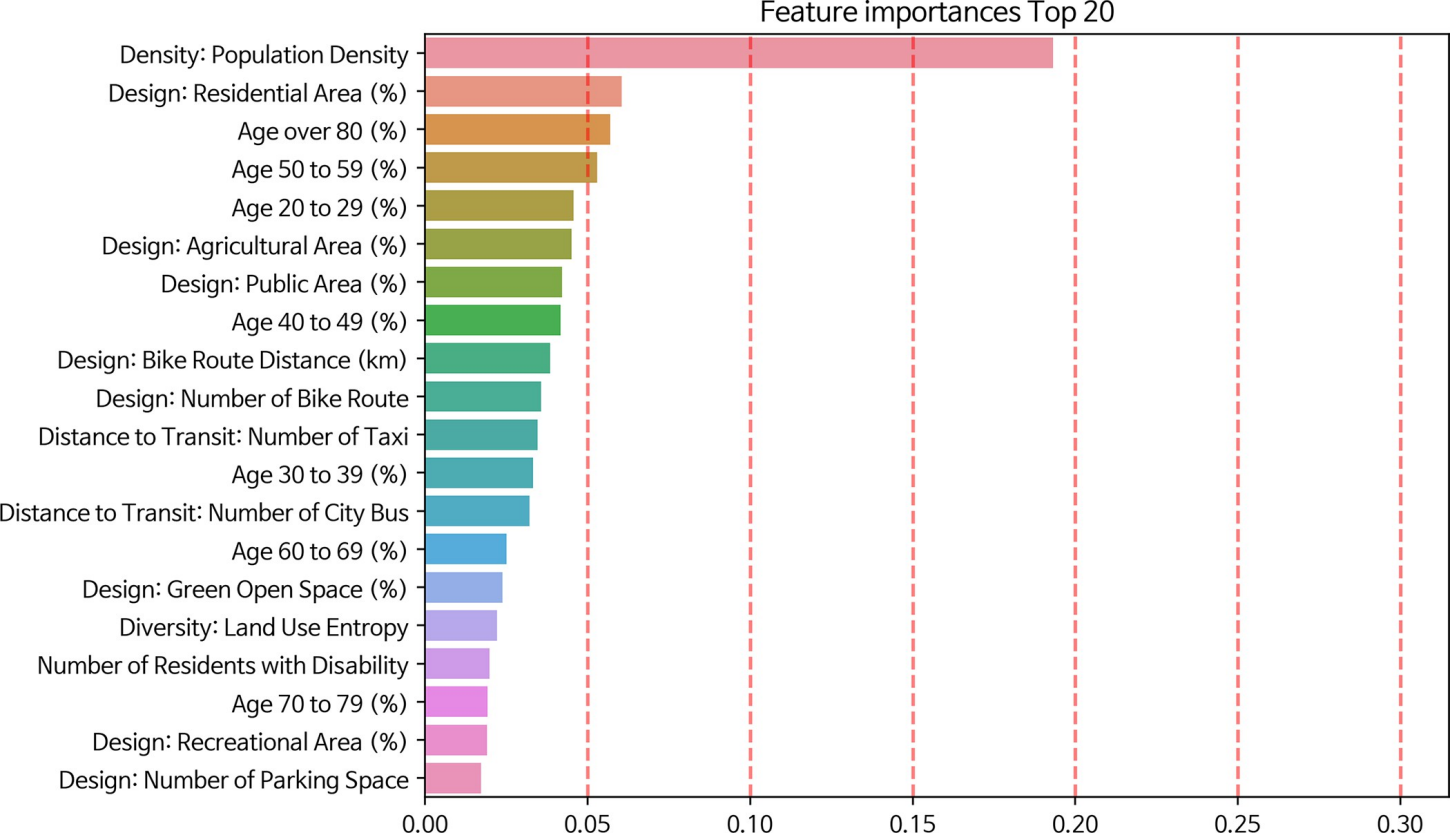
Feature importance in trained Random Forest model.

Compared to SVM, KNN, and DT, our RF model achieved the highest accuracy, precision, and F1 score. Although its recall score was lower than that of DT, RF had the fewest misclassified cases. Consequently, we chose to use RF. Fig 6 presents the top 20 features ranked by importance as extracted from the selected RF model. The result suggests that several factors play a significant role in training the classification model, including attributes related to density, design, distance to transit, diversity in the built environment, and sociodemographic characteristics. The attribute concerning population density emerged as the most influential. Design attributes associated with land use patterns, which account for the probability of areas being designated as residential, agricultural, public, green open space, recreational, and the overall land use entropy, were also paramount. Other noteworthy features that played a pivotal role during the training include the distance of bike routes, the number of bike routes, and the availability of parking spaces. Specific transportation modes, such as the number of taxis and city buses, were highlighted as highly influential. Lastly, sociodemographic elements, such as the proportion of residents across different age groups and the count of residents with disabilities, were identified as crucial in influencing the classification’s outcomes.

A deeper analysis of the specific feature directions for classifying transit deserts and oases was undertaken using DICE and SHAP value calculations. We employed DICE and SHAP for local validation of transit desert classifications and to assess the potential impact of machine learning on transit desert analysis, aiming to understand how specific feature influences contribute to model predictions on an individual basis. Table 2 summarizes the diverse counterfactual scenarios where transit deserts are envisioned as transit oases in hypothetical “what-if” situations. The scenarios often presented extreme changes, such as a significant reduction in population density and a higher proportion of green open spaces. Specifically, a transition from a transit desert to a transit oasis is possible when the population density decreases tenfold. The projected green open space more than doubled, increasing from 39% to 84%. As the registered census population decreased, there was a corresponding decline in the ratio of different age groups and the number of disadvantaged groups. The projected numbers of elderly living alone, low-income households, and residents with disabilities changed to 875, 1,211, and 754, respectively, from initial counts of 1,290, 978, and 1,149. On the supply side, improvements were observed in factors like bike route distance, the number of parking lots, bike routes, and transit stops, along with an increase in the number of city buses and bus stops. The projected number of parking lots increased by 6%, the number of bike routes rose from one to five, and the total number of transit options increased significantly from 26 to 68, including a substantial increase in city buses from 6 to 45 and bus stations from 17 to 61. Also, the projected number of taxis significantly increased from 110 to 770. However, the projected number of metro stations remained unchanged. The projected number of bike stations decreased from seven to five.

**Table 2:** DICE result.

Variable	Original	Diverse Counterfactual Set: From Transit Desert to Transit Oasis				
Real-time Metro Users	323,110	189,380	96,913	91,481	95,560	108,691
Real-time Bus Users	11,844	33,306	26,226	25,163	22,582	19,484
Real-time Bike Users	510	246	179	177	176	166
Density: Population Density	29,686	2,780	2,780	2,780	2,780	2,780
Diversity: Land Use Entropy	0.15	0.06	0.06	0.06	0.06	0.06
Design: Road Ratio (%)	0.20	0.02	0.02	0.02	0.02	0.02
Design: Bike Route Distance (km)	1	8	8	8	8	8
Design: Number of Parking Lot	1,218	1,291	1,291	1,291	1,291	1,291
Design: Number of Parking Space	10,372	7,680	7,680	7,680	7,680	7,680
Design: Number of Bike Route	1	5	5	5	5	5
Design: Residential Area (%)	0.21	0.02	0.02	0.02	0.02	0.02
Design: Industrial Area (%)	0	0	0	0	0	0
Design: Commercial Area (%)	0.11	0.02	0.02	0.02	0.02	0.02
Design: Recreational Area (%)	0.01	0.00	0.00	0.00	0.00	0.00
Design: Transportation Area (%)	0.00	0.00	0.00	0.00	0.00	0.00
Design: Public Area (%)	0.04	0.04	0.04	0.04	0.04	0.04
Design: Agricultural Area (%)	0.01	0.00	0.00	0.00	0.00	0.00
Design: Green Open Space (%)	0.39	0.84	0.84	0.84	0.84	0.84
Distance to Transit: Total Number of Transit	26	68	68	68	68	68
Distance to Transit: Number of City Bus	6	45	45	45	45	45
Distance to Transit: Number of Taxi	110	770	770	770	770	770
Distance to Transit: Number of Bike Station	7	5	5	5	5	5
Distance to Transit: Number of Bus Station	17	61	61	61	61	61
Distance to Transit: Number of Metro Station	2	2	2	2	2	2
Age 0 to 9 (%)	0.04	0.02	0.02	0.02	0.02	0.02
Age 10 to 19 (%)	0.06	0.03	0.03	0.03	0.03	0.03
Age 20 to 29 (%)	0.43	0.22	0.22	0.22	0.22	0.22
Age 30 to 39 (%)	0.26	0.18	0.18	0.18	0.18	0.18
Age 40 to 49 (%)	0.14	0.12	0.12	0.12	0.12	0.12
Age 50 to 59 (%)	0.14	0.12	0.12	0.12	0.12	0.12
Age 60 to 69 (%)	0.12	0.09	0.09	0.09	0.09	0.09
Age 70 to 79 (%)	0.08	0.05	0.05	0.05	0.05	0.05
Age over 80 (%)	0.03	0.03	0.03	0.03	0.03	0.03
Number of Elderly Living Alone	1290	875	875	875	875	875
Number of Low-Income Household	978	1211	1211	1211	1211	1211
Number of Residents with Disability	1149	754	754	754	754	754

**Fig 7:**
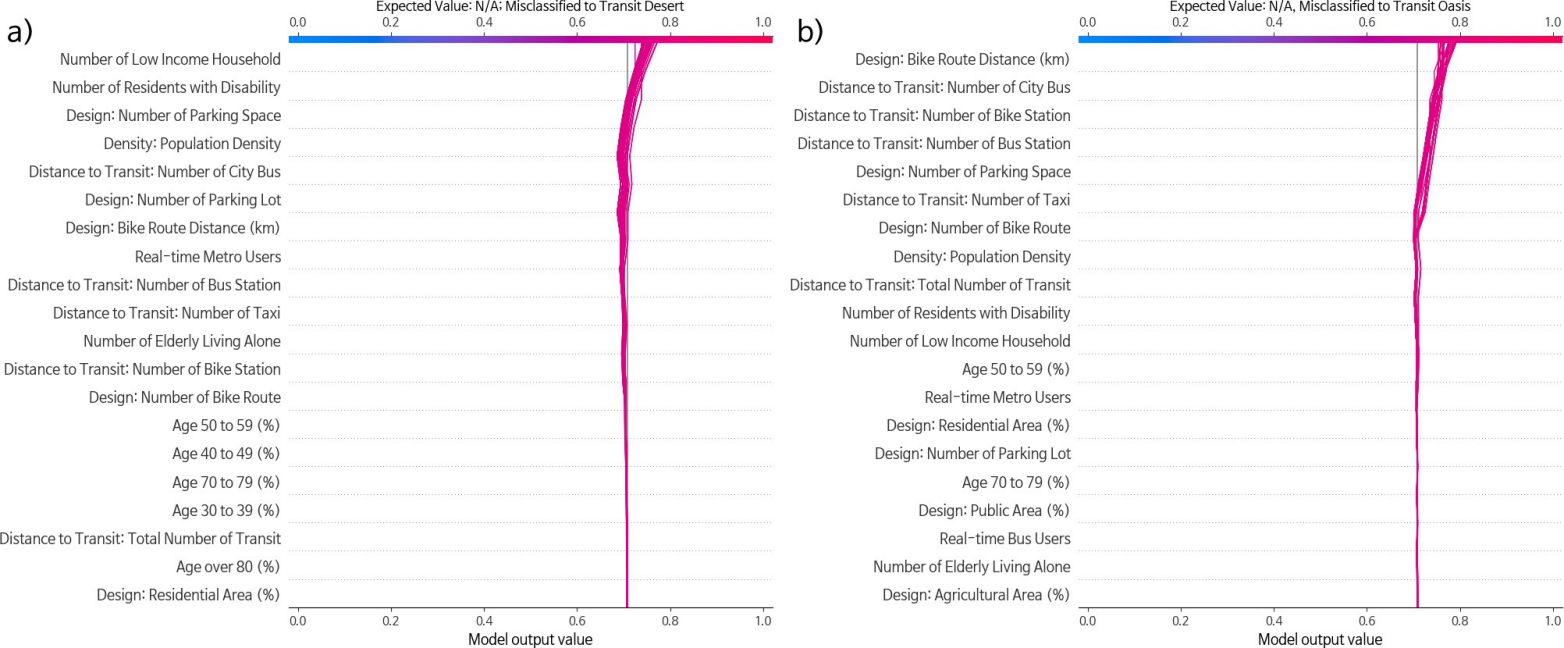
(a) Decision plot for samples misclassified as transit desert. (b) Decision plot for samples misclassified as transit oasis.

Fig 7 illustrates how the trained and selected RF model estimates the influence of specific features on the classification. This is simplified by focusing on misclassified samples. Misclassified instances of transit deserts and transit oases are depicted in Fig 7A and 7B, respectively, using SHAP’s decision plots. The findings suggest that the SHAP value remains relatively stable in the scenarios for transit deserts, not deviating considerably from the base value until it encounters socio-demographic data. This includes metrics such as the number of residents with disabilities and low-income households. In the scenarios for transit oases, the model perceives transit supply attributes as having a significant influence on the increased probability of an area being categorized as a transit oasis. These attributes encompass the number of taxis, parking spaces, bus stations, bike stations, city buses, and the distance of bike routes.

### 3.3. Localized validation of transit desert

**Fig 8:**
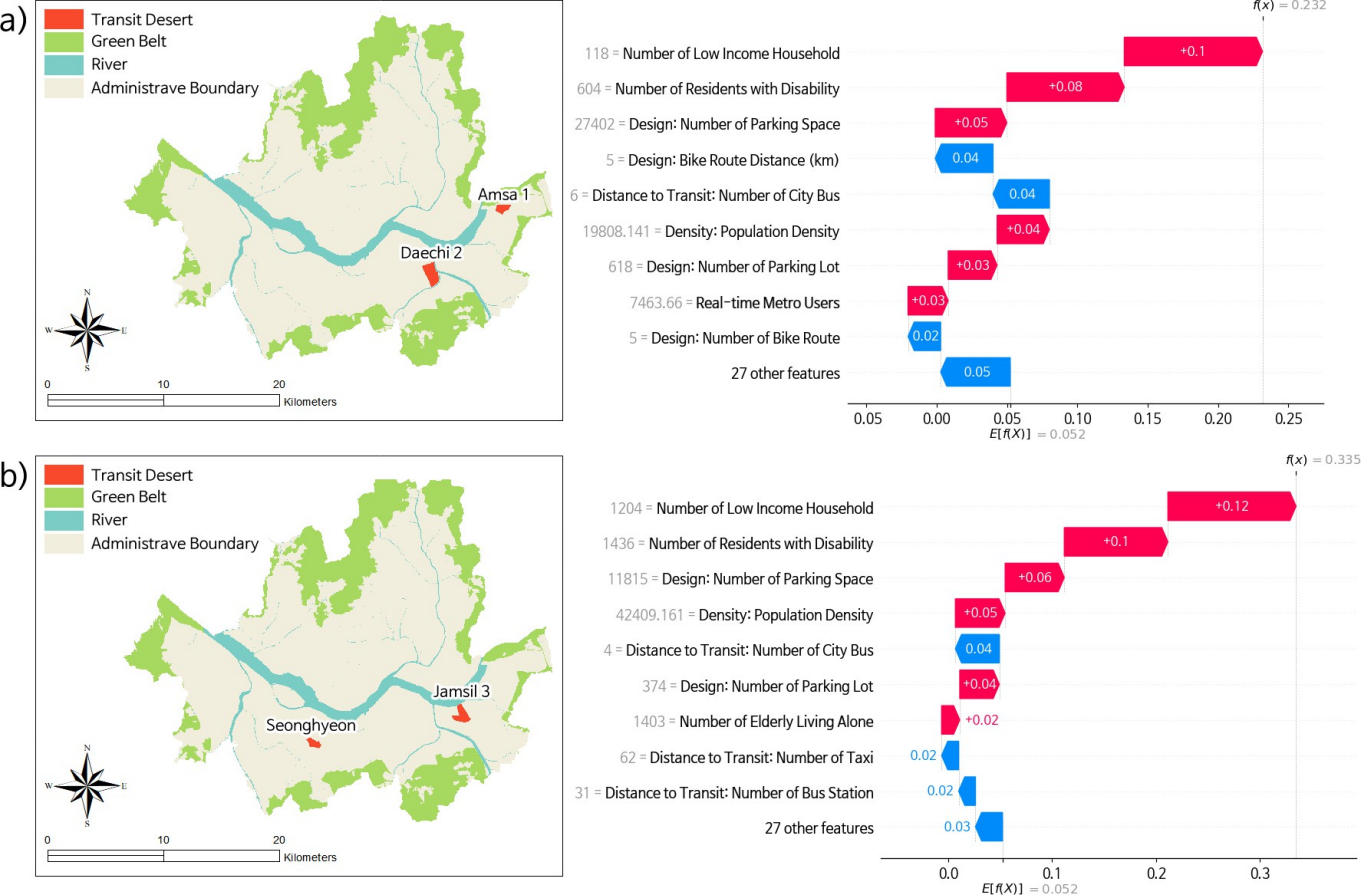
(a) Investigation of transit desert measured with male’s transit demand. (b) Investigation of transit desert measured with female’s transit demand.

Fig 8 delves into the validation of localized scenarios. The global SHAP cases are reintegrated with transit deserts, only evident in disaggregated cases, as visualized using a waterfall plot. Two distinct transit deserts were identified, each exhibiting unique characteristics not present in aggregated cases or specific to a gender. Both of these transit desert cases are predominantly located in the southern part of the city. The findings suggest that there is indeed a unique aspect to transit deserts that manifests differently between men and women. Fig 8A illustrates the case of a transit desert affecting men. Fig 8B depicts a transit desert affecting women. In the case of men, the two transit deserts identified were within the Amsa 1 and Daechi 2 administrative boundaries ("Dong" in local terms). For women, the transit deserts identified were within the Seonghyeon and Jamsil 3 administrative boundaries (“Dong” in local terms).

Consistent with the global estimation presented in Fig 7A, both cases imply that the number of low-income households and households with disabilities positively correlates with the chances of an area being classified as a transit desert. However, the extent to which each feature contributes varies. In the case of men, the number of low-income households contributed 10%, and the number of residents with disabilities contributed 8% to the likelihood of being classified as a transit desert. In the case of women, the number of low-income households contributed 12%, while the number of residents with disabilities contributed 10% to the likelihood of being classified as a transit desert. Transit deserts across different scenarios manifest under varying conditions. For instance, factors like bike route distance (-4%), the number of city buses (-4%), and bike routes (-2%) diminish the likelihood of an area being considered a transit desert in the case of men (as shown in Fig 8A). Conversely, the number of city buses (-4%), taxis, (-2%), and bus stations (-2%) decreases the probability in the case of women (as presented in Fig 8B). Additionally, elderly individuals living alone in the latter scenario slightly increase the classification probability by 2%. An increase in population density and the number of parking spaces each raised the likelihood by 4–5% and 5–6%, respectively, in both cases.

## IV. Discussion

This study introduced a supervised machine learning approach to predicting transit deserts. While primarily drawing from the research that explored the spatiotemporal identification of transit deserts in Seoul by Lee et al. [12], we examined transit deserts during peak periods. We employed a backpropagation approach for output classification. Both aggregated demand and gender-disaggregated demand were considered during the sample validation process. Our primary objective was to progress beyond just identifying transit deserts. We aimed to bridge the gap from identification to mitigation, linking transit desert analysis with transportation equity and challenging the oversimplifications commonly found in transportation equity literature.

The findings of our study are as follows. First, analysis results concerning transportation equity differ when using aggregated versus disaggregated data. When disentangling transit demand factors according to different sexes, cases of transit deserts and transit oases only appear in specific scenarios. It’s important to note that both transit deserts and transit oases signify imbalances between transit demand and supply. Women experience a greater mismatch, evident from a higher number of transit deserts and transit oases compared to the results from aggregated data and men’s demand. The differences highlight that transportation analysis should account for the fundamental differences in travel behavior among individuals [16,30], in addition to the variations observed when different methodologies are employed [2].

Through the lens of equity, it’s imperative to understand that the capability and availability of transportation vary among individuals. Commonly, women and gender minorities face harassment while using public transit [15,51]. Planning practices aimed at reducing disparities among specific community groups should steer clear of oversimplifying scenarios through aggregation. Simple normative assumptions about race, gender, and sexuality should not shape urban planning [52]. As evidenced in the characteristics of unique transit deserts compared to aggregated cases (refer to Fig 8), planning policies should aim to address distinct existing needs. While it is crucial to prioritize the requirements of disadvantaged groups, efforts to mitigate transit deserts should be tailored to contemporary conditions. Such measures would inherently differ when enhancing the factors of transit supply.

Second, we successfully modeled the classification of transit deserts and transit oases using a supervised framework. The emergence of AI-affiliated toolkits, including machine learning and deep learning, has garnered significant interest among researchers. The challenge now is determining the best ways to utilize new methods. Our research sought to merge a branch of transportation theory with new methodologies. In particular, we integrated transportation equity literature with the concept of transit deserts. We compared multiple multi-class classification machine learning models and found the RF model outperforming the rest. The selected RF was notably effective in identifying regions as transit deserts, N/A, or transit oases (refer to Figs 2 and 6). Furthermore, the model captured the characteristics of transit deserts and transit oases in Seoul. As depicted in Fig 7, supply dimensions were closely tied to transit oases, whereas the presence of disadvantaged groups correlated strongly with the likelihood of regions being classified as transit deserts [12].

**Fig 9:**
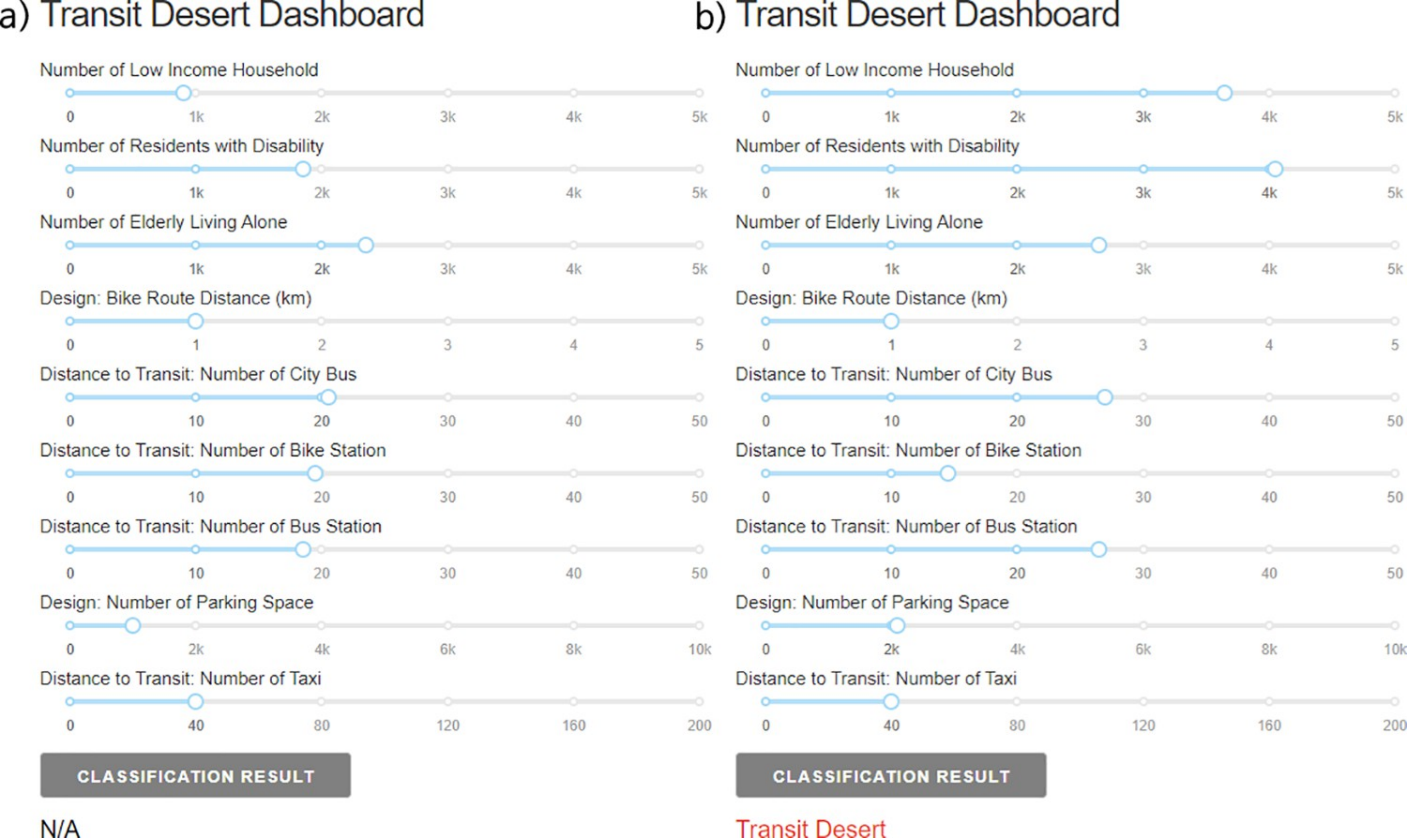
(a) Demonstration of the transit desert dashboard displaying an N/A case; (b) Demonstration of the transit desert dashboard displaying a transit desert case.

A potential application of our model is to integrate it into a dashboard or interactive toolkit, with the model functioning in the background. For instance, pre-trained models can be saved in a.sav format and later imported using the pickle module in a Python environment. Such an approach can facilitate the development of an interactive dashboard, as illustrated in Fig 9. Our dashboard demonstration offers a simplified method for altering the numeric values of significant variables. As users modify these inputs, the classification result updates in real-time. To illustrate, comparing the classification result labeled as N/A in Fig 9A. with Fig 9B. shows that increasing the number of disadvantaged individuals prompts the model to reclassify the region as a transit desert. Drawing parallels with the crowdsourcing toolkit [53], using this model in conjunction with community outreach, engagement, and participatory planning practices could allow residents to better understand and influence the characteristics of their communities.

Tracing back through planning history, the evolution of equity planning has been a gradual response to societal and economic factors [54]. Its origins can be traced to advocacy planning in the 1960s and 1970s, which marked a pivotal shift from the physical realm of planning towards addressing pressing social and economic issues, such as urban poverty and unemployment [54]. The history of segregation, characterized by redlining and exclusionary zoning, marginalized low-income working-class individuals and people of color. This necessitated a shift where planners should advocate for marginalized communities in pursuit of public goods.

Equity planning has evolved gradually, influenced by historical events and broader societal and economic factors [54]. Reece [54] well documented the evolution of equity planning. During the Progressive Era, equity planning responded to issues such as immigration, industrialization, the emergence of tenements, and increasing inequality. The New Deal era prompted responses to address the Great Depression. Advocacy planning emerged in response to the Civil Rights Movement, the legacy of urban renewal, and social conflict. The Just City era addresses challenges related to globalization, immigration, rising inequality, and gentrification. Given that equity planning aims to pursue social justice and advocate for the needs of the marginalized, its core essence remains consistent.

Transportation equity aligns with traditional equity planning paradigms, where the goal is an equitable redistribution of transportation resources. However, a critical perspective suggests that advocacy planners, despite their intentions, may exhibit political naivety and engage in tokenistic practices without yielding actual outcomes. This has led some scholars to critique the employment of ’equity’ as mere lip service [3,35], without effecting meaningful changes in the decision-making process and its deliberations.

A foundational case in equity planning from Cleveland by Krumholz [55] suggested that planners should leverage the power of information, analysis, and insight with an equity-focused lens in the decision-making process. However, the challenge lies in overcoming the status quo and ensuring the planning process remains truly informative. Emphasizing community participation and striving to be genuinely ’informative’ are identified as critical to the success of equity planning. With the advent of AI and related technologies, equity planning is poised for the next evolutionary leap. Our model introduces AI-augmented planning with a ’planner-on-the-loop’ approach [56]. Here, the focus remains on planners maintaining a strong lens of equity in planning. The integration of AI, transitioning from an ’AI out of the loop’ to an ’AI in the loop’ approach, refines planning practices and policies. Our empirical approach exemplifies how, in addressing issues such as transit deserts, transportation equity planning can progress to a more advanced stage while incorporating classic planning principles.

John Forester [57], who has significantly influenced contemporary planning practices with his communication action theory, highlights information as a source of power. He asserts the planner’s role in addressing information and anticipating misinformation while communicating with various stakeholders in the decision-making process. Communicative planners emphasize about crucial role in facilitating collaboration among stakeholders and affected groups through a creative process [58]. Their tools include various communication practices, such as listening, storytelling, rhetoric, mediation, and the use of metaphors. Now, with AI in the loop, contemporary communication practices are at a turning point.

An interactive dashboard fundamentally requires human auditing and management. The essence of a planner’s role while utilizing AI depends on how effectively planners communicate using AI or systems incorporating AI. This involves issues such as how we use AI, how we present it, and how it is employed in decision-making processes and outcomes. Historical epistemological debates in planning still resonate today.

Based on the theoretical framework of communicative action theorists, perhaps we should explicitly share the limitations of using the interactive dashboard. The fundamental dilemma of using our dashboard likely sustains criticism about biases in the dataset and errors in machine learning models [59]. How people react to decisions made by computational algorithms also varies depending on how they perceive the decision to be, whether less fair or trustworthy [60]. Planners, acting as facilitators or negotiators in the communication process, should strive to give equal opportunities to both speakers and listeners. If planners or developers are perceived as having more power or authority because they oversee the algorithmic decision-making system, we should then attempt to mitigate our inherent authority. Fjeld et al. [61] described accountability in AI as the anticipation that those who design, develop, and deploy AI systems will adhere to established standards and laws, ensuring AI operates correctly throughout its lifespan. Thus, the issue of AI systems compromising accountability lies with those who designed them. Situating value in the AI system needs further study.

Lastly, transit deserts continue to persist in Seoul. Due to changing travel behaviors, population movements, and the implementation of new planning policies, mismatches between transit demand and supply inevitably occur. If we analyze the identified transit deserts based on data primarily obtained from the Seoul Metropolitan Government database, transit deserts are areas where disadvantaged groups are more likely to reside. The result is similar to the transit desert analysis in Seoul conducted by Lee et al. [12]. The average number of elderly living alone, low-income households, and residents with disabilities in the transit deserts was 1,498, 1,172, and 1,191, respectively. These figures are higher than those in transit oases and regions that are neither. In transit oases, the averages for elderly living alone, low-income households, and residents with disabilities were 978, 659, and 896, respectively. For regions neither classified as transit deserts nor oases, these numbers were 832, 580, and 798, respectively. In the meantime, the average population density in transit deserts was the highest at 33,237 per km^2^, compared to 9,074 per km^2^ in transit oases and 6,445 per km^2^ in regions neither classified as transit deserts nor oases.

Throughout the study period, the average hourly numbers of metro, bus, and bike users in transit deserts were 48,241, 8,735, and 368, respectively. These figures are lower than those in transit oases, where the average hourly numbers for metro, bus, and bike users were 48,999, 9,074, and 713, respectively. Regions classified as neither transit deserts nor oases reported even lower mean values: 21,137 for metro, 6,445 for bus, and 309 for bike users. It was noted that the destination and distance to transit attributes of the built environment in transit deserts were insufficient compared to those in transit oases. On average, transit deserts had 44 total transit stops, 12 city buses, 145 taxis, 7 bike stations, 36 bus stations, and one metro station. In contrast, transit oases comprised 67 transit options: 62 buses, 570 taxis, 15 bike stations, 50 bus stations, and 2 metro stations. These findings suggest that public transit use in transit deserts is significant and is relatively comparable to use in transit oases. However, the destination and distance attributes of the built environment in transit deserts are less sufficient than those of transit oases. Moreover, transit deserts had the lowest average green open space at 22%, compared to 56% in transit oases and 32% in areas classified as neither.

Using disaggregated demand cases, two distinct transit deserts were identified for both men and women. In the former case, the Amsa 1 and Daechi 2 administrative boundaries (locally termed "Dong") were noted. In the latter case, the Seonghyeon and Jamsil 3 administrative boundaries fall into this category. The Amsa 1 administrative boundary reported a relatively high residential ratio of 42%. This region has only 4 bike stations and 13 bus stations and lacks a metro station, which is below average for typical transit desert cases. However, its population density is notably high at 46,137 people per km^2^. It also has a significant number of disadvantaged groups, with 1,714 elderly living alone, 1,351 low-income households, and 1,692 residents with disabilities. These figures exceed the average characteristics found in transit deserts. On the other hand, the Daechi 2 administrative boundary appears to have more family households with children, as the population aged 10 to 19 makes up 23%, and those aged 40 to 59 constitute 49% of its residents. This area has fewer disadvantaged community members, with relatively low numbers of elderly living alone (594), low-income households (118), and residents with disabilities (604).

The Seonhyeon administrative boundary exhibited a number of disadvantaged groups comparable to those in the Amsa administrative boundary. It reported 1,403 elderly living alone, 1,204 low-income households, and 1,436 residents with disabilities. The Seonhyeon administrative boundary has limited public transportation options, with only 4 city buses, 1 bike station, and no metro station. On the other hand, the Jamsil 3 administrative boundary is known for its high population density, at 55,929 per km^2^. It’s notable for recreational activities, with one of Seoul’s tallest buildings serving as a landmark. This area has 48% green open space and predominantly houses an affluent community. Consequently, there are only 44 low-income households, alongside 728 elderly living alone and 681 residents with disabilities.

For general improvements to mitigate the occurrence of transit deserts, enhancing the design and proximity of transit attributes within the built environment can be broadly applied. Specifically, DICE identified that green open spaces, tied to travel satisfaction [62], significantly increase the likelihood of a transit desert transforming into a transit oasis. Transit deserts were also confirmed to have less green open space compared to transit oases, or cases neither classified as deserts nor oases. Improvements to bike route distances, the number of parking lots, bike routes, transit stops, and the installation of additional bus lanes and stops could also play pivotal roles. They have been identified as mitigating factors that reduce the likelihood of regions being classified as transit deserts in SHAP analysis.

Additionally, it is crucial to note that communities’ individual characteristics and current statuses should be addressed. For instance, to address the transit needs within the Amsa 1 and Seonhyeon administrative boundaries, it is critical to consider the needs of disadvantaged groups. Public intervention is necessary to enhance the overall public transit services in Amsa 1 and Seonhyeon administrative boundaries. In the Daechi 2 administrative boundary, a transportation policy that caters specifically to the needs of family households appears to be more suitable. The approach for the Jamsil 3 administrative boundary should aim to support recreational trips and effectively manage the area’s comparatively high population density. We are not advocating for a one-size-fits-all planning intervention. Rather, during the decision-making process, when evaluating alternatives with incremental differences, considering the unique characteristics of each region can help planners and policymakers make better decisions.

Lastly, transit deserts exist around the globe [1,11,12,22]. The mismatch between transit demand and supply is inevitable. The focus should be not on solving the problem entirely but rather on mitigating the occurrence of transit deserts. The case study of Seoul in S. Korea can be applied to other cities to develop a forecast model to address the causes of transit deserts and advocate for the needs of disadvantaged groups. However, similar to our efforts in addressing localized scenarios, what constitutes transit supply and demand factors should be contextualized. The level of service in transit differs depending on the size of the city. Metropolitan regions, megaregions, urban, suburban, and rural areas have different conditions. The case study of Seoul mainly falls into the category of studying a metropolitan region with a high population density. We do not suggest directly applying the analysis of transit deserts without modifications.

## V. Conclusions

The present study outlines a supervised research framework for modeling transit desert classification using machine learning. We utilized three class labels: Transit Desert, N/A, Transit Oasis by comparing transportation demand with supply factors. We evaluated several algorithms: SVM, DT, RF, and KNN, and performed hyperparameter optimization. Based on the evaluation metrics, we chose RF. We extracted feature importance from the trained RF model. Both global and local validations of identified transit deserts and the impact of collected features on classification probabilities were analyzed using DICE and SHAP values. Later, we integrated the trained RF model to demonstrate its practical application in developing interactive interface.

Our research offers several contributions to the field. Primarily, we expand on existing studies about transit deserts, moving beyond mere investigation to develop a modeling framework that addresses the multifaceted factors associated with transit deserts. Our identification of transit deserts aligns with existing transit desert analyses [12]. Transit deserts are regions where a larger number of disadvantaged groups are located and where the supply of transit is insufficient. Transit desert analysis is a needs gap analysis that quantifies transportation poverty within the scope of transportation equity analysis [63]. However, the challenge lies in the criteria selected for this analysis [63]. It remained uncertain how the results of transit desert analysis might vary should we disaggregate the aggregated transit demand by gender.

In our approach to identifying transit deserts, we differentiated the outcomes of transportation equity analyses by comparing aggregated data with disaggregated data (separating demands between men and women). Our study emphasized the importance of contextualizing planning efforts. We found unique transit deserts for both men and women, showing that results change when we look at aggregated data versus disaggregated data. The transit deserts we identified each have their own set of needs. Some require more focus on helping disadvantaged groups, another needs more support for families with children, and another should concentrate on improving options for recreational trips.

Moreover, we evaluated multiple machine learning models, culminating in the demonstration of a transit desert dashboard. The interactive dashboard incorporates human intervention into transportation equity analysis. By adjusting the threshold values, users can determine whether their region is identified as a transit desert. Algorithms and AI systems with machine learning become more effective when centered around human needs. Human intervention in the decision-making process helps users trust the outcomes generated by machines [64]. Creating an interactive dashboard differs from a static information viewer dashboard. It opens up new avenues for innovative participatory planning practices. It adds a new tool for communicative action planners, especially. We showcased the case of transportation poverty using a needs-gap analysis approach, but other tools in the planning equity analysis toolkit can also be designed with AI systems. Ultimately, no models are perfect, and each method has its limitations. Holistically combining multiple sets of tools and using them as both means and for outcomes might be beneficial.

However, sustained criticism exists about biases and errors in computational algorithms [59]. Future studies should consider exploring the discrepancies between identification by AI and residents’ perceptions in defining their communities, which would validate different use cases. Therefore, qualitative research involving user surveys, interviews, and data auditing is necessary.

Future research might also benefit from focusing on specific analyses of green transportation needs. This focus is critical because transportation significantly contributes to greenhouse gas emissions, a major factor in climate change that we need to mitigate. We are increasingly experiencing the impact of climate hazards in our daily lives, leading to the use of the term ’climate extremes’ to describe the severe consequences of climate change. The role of green transportation, such as electrifying transport systems and integrating them with renewable energy, is gaining recognition for its importance. The analysis of transportation equity is linked to environmental justice [3], seeking to fairly address the unequal/disproportionate impacts of emerging issues across different communities. There could be disparities in how green transportation is adopted and accessed. Identifying areas lacking in green transportation, or ’green transit deserts,’ can aid in establishing a baseline that tackles the disparities in the adoption of green transportation.

However, it is essential to acknowledge several limitations. Our study primarily focuses on transit deserts during peak-time periods. Supervised machine learning, on its own, can only be optimized by being fine-tuned based on future data insights. While our suggestions for mitigating transit deserts primarily focus on improvements on the supply side of transit, the influence of individual attributes should not be overlooked. Involving machines, or AI, in decision-making requires building trust and situating value in the AI system. This involves enhancing the system’s accountability and mitigating biases in the datasets used for training the machines [59]. Communicative planners hold public meetings, town hall meetings, focus group meetings, charrettes, Delphi methods, and other forums to communicate and build trust. How interactive dashboards can be applied to different types of communicative planning tools remains a critical implementation challenge. We earmark these areas for future research.

## Supporting information

S1 Table Descriptive statistics. (DOCX)
