# Supplementary material for: Developing a transit desert interactive dashboard: Supervised modeling for forecasting transit deserts
Source: PLoS One. 2024 Jul 24;19(7):e0306782. doi: 10.1371/journal.pone.0306782 (PMC11268652; doi:10.1371/journal.pone.0306782)
Supplement: S1 Table — (DOCX) [file pone.0306782.s001.docx]

**Appendix**

**S1 Table**. Descriptive Statistics^[[1]](#footnote-1)^

| **Variable** | | **mean** | **std** | **min** | **median** | **max** |
| --- | --- | --- | --- | --- | --- | --- |
| Aggregated  Transit Demand | Aggregated Human Dynamics | 8,932,226 | 4,728,787 | 1,277,797 | 7,790,049 | 40,249,993 |
|  | Census Population between age 20 and 84 | 18,691 | 7,487 | 19 | 18,081 | 46,153 |
|  | Registered Vehicle Number | 6,211 | 6,070 | 352 | 4,344 | 44,071 |
| Disaggregated  Transit Demand of Man | Male  Human Dynamics | 4,242,559 | 2,376,783 | 658,734 | 3,698,581 | 21,167,657 |
|  | Census Population of male between age 20 and 84 | 9,031 | 3,595 | 10 | 8,712 | 22,406 |
|  | Registered Vehicle Number × ratio of male between age 20 and 84 to total census population between age 20 and 84 | 3,009 | 2,950 | 173 | 2,098 | 22,280 |
| Disaggregated  Transit Demand of Woman | Female  Human Dynamic | 4,689,666 | 2,391,229 | 619,063 | 4,167,731 | 19,082,335 |
|  | Census Population of female between age 20 and 84 | 9,659 | 3,922 | 9 | 9,291 | 23,746 |
|  | Registered Vehicle Number × ratio of female between age 20 and 84 to total census population between age 20 and 84 | 3,201 | 3,138 | 179 | 2,241 | 23,662 |

| Transit  Supply | Destination:  Real-time  Metro Users | 24,186 | 52,239 | 0 | 0 | 1,115,350 |
| --- | --- | --- | --- | --- | --- | --- |
|  | Destination:  Real-time  Bus Users | 6,719 | 6,736 | 0 | 5,551 | 133,294 |
|  | Destination:  Real-time  Bike Users | 336 | 556 | 0 | 182 | 16,241 |
|  | Density:  Population Density | 22,208 | 11,036 | 26 | 22,297 | 50,299 |
|  | Diversity:  Land Use Entropy | 0.14 | 0.03 | 0.04 | 0.14 | 0.24 |
|  | Design:  Road Ratio (%) | 0.15 | 0.06 | 0.01 | 0.14 | 0.33 |
|  | Design:  Bike Route Distance (km) | 2 | 3 | 0 | 2 | 24 |
|  | Design:  Number of  Parking Lot | 723 | 526 | 9 | 625 | 3,162 |
|  | Design: Number of Parking Space | 10,618 | 7,250 | 193 | 8,622 | 53,019 |
|  | Design: Number of Bike Route | 2.95 | 3.40 | 0.00 | 2.00 | 31.00 |
|  | Diversity:  Land Use Entropy | 0.14 | 0.03 | 0.04 | 0.14 | 0.24 |
|  | Design:  Residential Area  (%) | 0.19 | 0.12 | 0.01 | 0.17 | 0.50 |
|  | Design:  Industrial Area  (%) | 0.00 | 0.01 | 0.00 | 0.00 | 0.12 |
|  | Design:  Commercial Area (%) | 0.10 | 0.08 | 0.01 | 0.09 | 0.56 |
|  | Design:  Recreational Area (%) | 0.01 | 0.01 | 0.00 | 0.01 | 0.07 |
|  | Design: Transportation Area (%) | 0.02 | 0.04 | 0.00 | 0.00 | 0.38 |
|  | Design:  Public Area (%) | 0.03 | 0.02 | 0.00 | 0.03 | 0.15 |
|  | Design:  Agricultural Area (%) | 0.01 | 0.02 | 0.00 | 0.00 | 0.23 |
|  | Design:  Green Open Space (%) | 0.33 | 0.20 | 0.02 | 0.30 | 0.89 |
|  | Distance to Transit: Total Number of Transit | 37 | 22 | 1 | 32 | 164 |
|  | Distance to Transit: Number of City Bus | 17 | 26 | 0 | 11 | 279 |
|  | Distance to Transit: Number of Taxi | 169 | 215 | 2 | 115 | 1,982 |
|  | Distance to Transit: Number of Bike Station | 6 | 6 | 0 | 5 | 48 |
|  | Distance to Transit: Number of Bus Station | 30 | 18 | 1 | 26 | 138 |
|  | Distance to Transit: Number of Metro Station | 1 | 1 | 0 | 1 | 5 |
| Socio-demographic Information  from Census | Census Population: Age 0 to 9  (%) | 0.05 | 0.03 | 0.00 | 0.04 | 0.24 |
|  | Census Population: Age 10 to 19 (%) | 0.07 | 0.04 | 0.00 | 0.06 | 0.27 |
|  | Census Population: Age 20 to 29 (%) | 0.12 | 0.06 | 0.00 | 0.11 | 0.43 |
|  | Census Population: Age 30 to 39 (%) | 0.12 | 0.06 | 0.00 | 0.12 | 0.39 |
|  | Census Population: Age 40 to 49 (%) | 0.13 | 0.06 | 0.00 | 0.12 | 0.36 |
|  | Census Population: Age 50 to 59 (%) | 0.13 | 0.05 | 0.00 | 0.12 | 0.32 |
|  | Census Population: Age 60 to 69 (%) | 0.11 | 0.05 | 0.00 | 0.11 | 0.27 |
|  | Census Population: Age 70 to 79 (%) | 0.06 | 0.03 | 0.00 | 0.06 | 0.15 |
|  | Census Population: Age over 80  (%) | 0.03 | 0.01 | 0.00 | 0.03 | 0.07 |
|  | Number of Elderly Living Alone | 996 | 508 | 2 | 884 | 3,145 |
|  | Number of Low-income Household | 681 | 515 | 1 | 572 | 4,199 |
|  | Number of Residents with Disability | 921 | 491 | 3 | 818 | 3,649 |

1. Real-time data is based on the monthly average during peak-time periods (12 hours) for each respective administrative boundary. The study period is set in 2022. All data are publicly accessible. [↑](#footnote-ref-1)
